# The Individual and the Homestead

**Published:** 1919-06-14

**Authors:** 


### The Hospital

The Workers' Newspaper of Administrative Medicine and Institutional Life, Administration, National Insurance and Health.

No. 1723, Vol. LXVI. SATURDAY, JUNE 14, 1919. PRICE TWOPENCE

### THE INDIVIDUAL AND THE HOMESTEAD.

Throughout the war we have taken occasion more than once to urge that if the British are in deed and in truth a great people the conclusion of the war will mean the creation of a real, living, splendid Merrie England for all classes. The existing conditions of life for many residents in Great Britain and Ireland cause large masses of the population to be threatened by grievous dangers to health, to life, and to much that makes life on this planet best worth living. Such a state of affairs cannot and must not survive an avoidable hour longer than the promptest, spontaneous and united action of the people of these islands can provide, by changing all these things and making them once for all worthy of the land of the free, of the Mother-country of many nations of English-speaking stocks, who, with India, are directly responsible for one-third of the globe. Alas! that we, as a nation, have permitted large masses of the population to grow up who, unaided, are unable in existing conditions to have to the fullest possible extent the health-giving advantages, the wholesome surroundings, the facilities for enjoying life and freedom, the comforts of the home, which are essential to enable them to live a full life in pursuance of Christ's teaching, and the upholding of the principles on which rest most things which make the doctrine of immortality desirable, and the spiritual life of the individual attainable to the full. Every one of the children of free England in the near future must not only be free, but be placed in a position to live his or her life in full vigour and blessedness, subject only to the conditions which will make this possible through the immediate action of the inhabitants of every centre, acting through the county, municipal, and local authorities, with the encouragement and aid of the Government.

The latest reports indicate, and there is sound evidence to believe, that Peace will be established by the end of July. There is, therefore, no time to lose for the active spirits in each community, in co-operation with or by direct pressure from the people in every centre throughout Great Britain, to organise and prepare for the setting up of the necessary machinery to secure the provision of real homes in buildings of the highest hygienic efficiency. Each full of facilities for the preservation of health, the enjoyment of life, the maintenance of moral laws, and the cheer and brightness without which, home and family life, in Christ's meaning of the words, are impossible. The Hammersmith Borough Council, to its eternal honour, has decided to lead by example this organisation of the people locally, to their own uplifting, by carrying out an enterprising scheme in municipal housing reform. The Council's plan involves the reconstruction of practically the whole riverside front from Fulham to Chiswick, and the reclamation of the large and overcrowded area from slum conditions to those of a communal modern town. The *Times* states that the improvements will embrace one of the oldest parts of London, and some still picturesque buildings will not be interfered with. The Borough Council is asking powers from the London County Council to proceed with the programme of demolishing all the inferior property of the riverside, and replacing it by open squares and gardens, to be laid out as a middle- and working-class residential suburb. This will be provided with central lighting and heating, both by electricity, a central laundry, probably a communal bakery, a local crêche, social clubs, and public-houses of modern design. It will be noted that the Hammersmith Council is resolved, with the co-operation of the inhabitants, to settle the lines of development and uplifting in this centre for an indefinite time to come.

It is further notable and encouraging that the riverside area will be arranged for a diversity of occupations, part of the new houses consisting of self-contained tenements, and the remainder of single family residences. Housewives will be glad, as we are, to note that, the interior arrangements of these houses will be on lines, designed to reduce their work to the minimum. The inhabitants who are fortunate enough to reside in redeemed Hammersmith will be given facilities for enjoyment, and the means under attractive conditions for maintaining their health and physical development. An esplanade is to be constructed for the length of nearly a mile along the Thames-side, starting from Hammersmith Bridge. The interests of the rowing clubs about this bridge have been safeguarded by providing that the fullest boating facilities are not to be curtailed, but to be improved. The City Companies, who are large landowners in the sections of Hammersmith to be converted, are co-operating with the Borough authorities in furtherance of the scheme. Everything, therefore, promises well in this pioneer attempt to make, at least, one centre within the Metropolis, the combined efforts and energy of its inhabitants free from the evils and cruelties of the past, and to substitute for them a residential quarter adapted to the highest needs of all types of residents. This attempt to lead the Great Movement of Reconstruction should be followed by every centre throughout Great Britain with all possible energy and despatch. Then The Peace when it comes will be celebrated as it should be, and Merrie England may indeed be for generations the pride and happiness of every toiler within the Motherland.

